# *Scaptotrigona mexicana* Propolis from Totonacapan Region: Chemical Composition, Antioxidant and Antibacterial Activities

**DOI:** 10.3390/molecules30061370

**Published:** 2025-03-19

**Authors:** Blanca E. Rivero-Cruz, Maria Ema Rojas-Brandao, Adriana Correa-Benítez, Ingeborg Becker, Aurora Xolalpa-Aroche, José Delgado-Dominguez, J. Fausto Rivero-Cruz

**Affiliations:** 1 Facultad de Química, Universidad Nacional Autónoma de México, Ciudad Universitaria, Ciudad de Mexico 04510, Mexico; 317516760@quimica.unam.mx; 2 Facultad de Medicina Veterinaria y Zootecnia, Universidad Nacional Autónoma de Mexico, Ciudad Universitaria, Ciudad de Mexico 04510, Mexico; adrianac@unam.mx; 3 Facultad de Médicina, Universidad Nacional Autónoma de Mexico, Ciudad Universitaria, Ciudad de Mexico 04510, Mexico; becker@unam.mx (I.B.); josesoterod@gmail.com (J.D.-D.); 4 Centro de Innovacion para el Desarrollo Apícola Sustentable en Quintana Roo, Universidad Intercultural Maya de Quintana Roo, Jose Maria Morelos 77890, Mexico; aurora.xolalpa@uimqroo.edu.mx; 5 Investigadores por Mexico, Comisión Intersecretarial de Bioseguridad de los Organismos Genéticamente Modificados (CIBIOGEM-SECIHTI), Ciudad de Mexico 03940, Mexico

**Keywords:** stingless bee, beehive products, terpenes, antibacterial, antioxidants

## Abstract

The propolis produced by stingless bees is a complex mixture of natural sticky components mixed with soil or clay. Global research on propolis has focused on studying the biological and pharmacological properties and chemical composition of stingless bee propolis from Brazil, Indonesia, and other regions. However, studies of stingless bee propolis produced in Mexico are scarce. This study aimed to determine the chemical composition of the geopropolis of *Scaptotrigona mexicana* collected in the Totonacapan region and to evaluate its antioxidant and antibacterial activities. The phenolic contents of the ethanolic extract of the collected propolis ranged from 2.45 ± 0.03 mg GAE/g to 3.48 ± 0.56 mg GAE/g of dry extract. The total flavonoid content ranged from 0.69 ± 0.03 mg QE/g to 0.84 ± 0.009 mg QE/g of dry extract. The antioxidant activity of the ethanolic extracts was assessed via DPPH, ABTS, and FRAP assays. The minimum inhibitory concentration values exhibited by the ethanolic extract (>512 g/mL) for Gram-negative bacteria (*Pseudomonas aerugunosa* and *Phorphyromonas gingivalis*) were higher than those of Gram-positive bacteria. The stingless bee propolis extract showed the highest antibacterial activity against *Streptococcus mutans* (256 g/mL). Five known compounds, taraxeryl acetate (**1**), lupeol (**3**), cicloart-23-en-3β,25-diol (**5**), mangiferoic acid (**6**), and 5-(11’Z-heptadecenyl)-resorcinol (**7**), and two irresoluble mixtures of 3-*O*-acetyl-α-(2a) and 3-*O*-acetyl-β-amyrins (**2b**), and α- (**4a**) and -amyrins (**4b**), were identified by nuclear magnetic resonance spectroscopy and mass spectrometry. Additionally, 39 volatile compounds were identified via headspace-solid phase microextraction using the hyphenated gas chromatography coupled to mass spectrometry time-of-flight. The main volatile compounds detected include *trans*-α-bergamotene (8.15%), hexanal (7.17%), 2-heptanone (7.60%), and α-copaene (7.09%).

## 1. Introduction

Stingless bees are a large group of more than 600 species, described in study [1,2]. These bees are distributed in subtropical and tropical regions [3]. They are key pollinators in many ecosystems and produce high-value products, such as honey and propolis [4].

**Figure 1:**
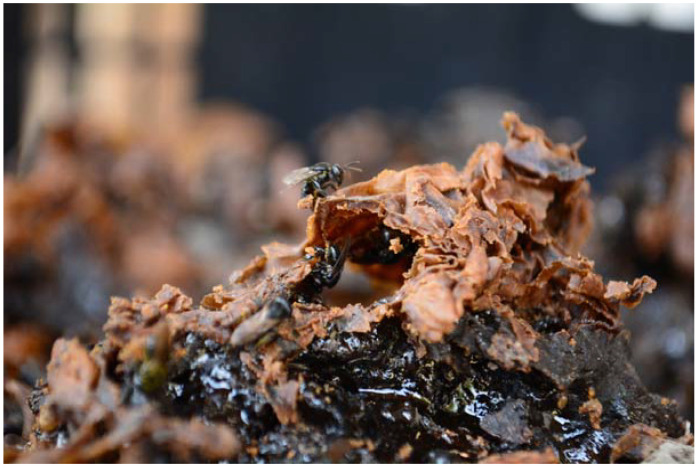
Scaptotrigona mexicana bee.

*Scaptotrigona mexicana* is a stingless bee species found in Mexico, mainly in Tamaulipas, Veracruz, Puebla, and Chiapas [5] (Figure 1). These bees are between 5.0 and 5.3 mm long and build their nests in tree trunk hollows with trumpet-shaped entrances. It is common to see individuals guarding the entrance [5,6]. This species is locally known as “abeja puerca”, “taxkat”, “Chinchin”, “abeja de tierra”, “enreda pelo”, “abeja de monte”, “toritos”, or “tanchalita”, “Pisilnekmej”, and “Congo negra” (Chiapas and Guatemala) [5,7].

Propolis is created by stingless bees from resins collected from plants, wax, and salivary gland secretions [8]. Bees use it to polish the internal walls, prevent mechanical damage, and seal holes or cracks in the hives. It also acts as a thermoregulatory agent to prevent exposure to drafts and is mixed with wax in nest construction, a material known as cerumen [3,6,8].

In traditional folk medicine, stingless bee propolis has been empirically used as an antibacterial agent and to treat respiratory and digestive diseases, visual problems, female fertility issues, and dermatosis [8,9]. Recently, it has been found to possess antinociceptive, anti-inflammatory, antiproliferative, antitumoral, anticancer, antioxidant, antibacterial, antiviral, antifungal, antimutagenic, and gastroprotective properties and to treat cardiovascular disorders and coronavirus disease (COVID-19) [10]. Propolis activity is related to its chemical composition, which is highly complex and varies depending on the type of bee, climatic factors, and the collection site [11,12,13]. The chemical composition varies depending on the region of origin and the vegetal sources available for bees [12,14]. Chemical studies of stingless bee propolis have shown the presence of aromatic acids, alcohols, phenols, terpenoids, aliphatic acids, sugars, flavonoids, saponins, coumarins, and steroids [14,15]. Currently, the chemical composition and biological properties of stingless bee propolis produced in Mexico are poorly studied, and the focus is mainly on *Melipona beecheii* [8].

In this context and based on the insufficient data about the chemical composition and biological activities of *Scaptotrigona mexicana* propolis, this study aimed to characterize the chemical composition, antioxidant, and antibacterial activities and isolate the major components of the ethanolic extract of stingless bee propolis collected in the Totonacapan region of Mexico.

## 2. Results and Discussion

### 2.1. Chemical Profile of Ethanolic Extracts of EEP

The chemical profiling of the propolis extract was performed using thin-layer chromatography and nuclear magnetic resonance (NMR). For deeper insights into its composition, 10 mg of the extract was analyzed using NMR. The ^1^H-NMR spectrum revealed that the primary constituents were terpenoids and fatty acids, with aromatic compounds at much lower intensities. The presence of aromatic compounds was confirmed by several signals observed between 6.0 and 7.5 ppm.

Recently, Gerginova and colleagues examined the chemical composition of 24 propolis samples of the stingless bee *Scaptotrigona mexicana* from two meliponaries in two locations in Chiapas, southern Mexico. The chemical composition of the propolis samples was studied using NMR and GC-MS. The samples from both locations contained cycloartane-type triterpenes (cycloartenol, mangiferolic, and isomangiferolic acids) and the group of phenolic lipids, mainly cardols (alk(en)yl resorcinols), described as chemical markers of *Mangifera indica*. The propolis composition of the two locations demonstrated qualitative differences, indicating a specific choice of resins by the bees. The variations in the chemical composition of these propolis, produced by *S. mexicana* species from geographically close locations, support the assumption that bee species are not necessarily the most critical factor in determining the chemistry of the propolis [14].

### 2.2. Isolation of Compounds ***1***–***7***

**Figure 2:**
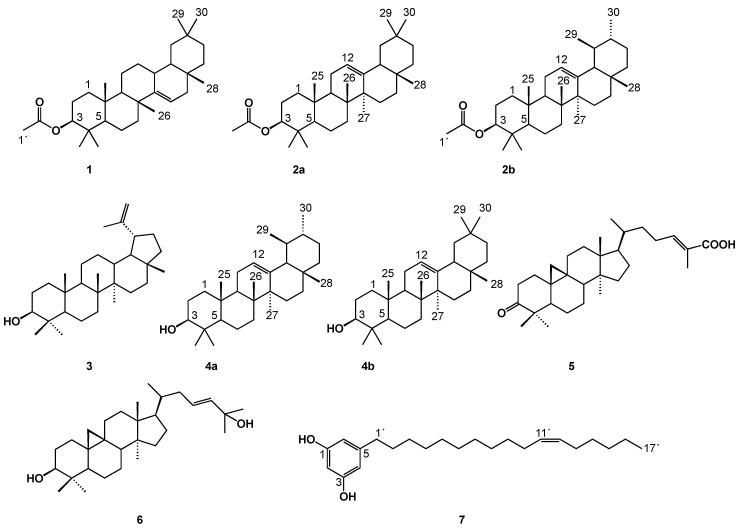
Compounds isolated from *Scaptotrigona mexicana* propolis.

The powdered stingless bee propolis (50.0 g) was extracted with 500 mL of ethanol three times with sonication at room temperature for 1 h, and the resultant extracts were concentrated under reduced pressure. Ethanol was selected for extraction as it is the most popular solvent for propolis extraction for medicinal uses [15] and replicating the traditional extraction method used in the Totonacapan region of Mexico for obtaining extracts with medicinal properties. This approach has enabled the analysis of the identity and content of the compounds obtained. The EtOH-soluble extract of stingless bee propolis was fractionated by chromatography on a VLC column, yielding five known compounds (**1**, **3**, and **5**–**7**) and two irresoluble mixtures (**2a**/**2b** and **4a**/**4b**). The compounds (Figure 2) were identified by employing NMR spectral analysis and by comparison with spectra found in the literature. The presence of taraxeryl acetate (**1**) [16], the mixture of 3-O-acetyl-α- and -β-amyrins (**2a**/**2b**) [17], lupeol (**3**), the α and β-amyrins (**4a**/**4b**) [18], cicloart-23-en-3β,25-diol (**5**) [19], mangiferoic acid (**6**) [20], and 5-(11’Z-heptadecenyl)-resorcinol (**7**) [21] suggests that its primary botanical sources are Bursera simaruba, Mangifera indica, and Lipia mexicana.

Although triterpenoids and sterols are commonly found in stingless bees’ propolis from Peninsula de Yucatan, this study is the first report of mangiferoic acid (**6**) and 5-(11’Z-heptadecenyl)-resorcinol (**7**) as constituents of stingless bee propolis collected in Mexico. To the best of our knowledge, 5-(11’Z-heptadecenyl)-resorcinol (**7**) had not been reported as a component of Scaprotrigona mexicana propolis. In a previous study of a stingless bee propolis produced by Melipona beecheii, 13 pentacyclic triterpenes, methyl-3-oxours-12-en-23-oate, marsformosanone, taraxerone, amyrenone, lupenone, 24-methylencycloartan-3-one, moretenol acetate, amyrin acetate, germanicol acetate, 24-methylencycloartanyl acetate, and amyrin were identified in a chloroform–methanol propolis extract. Other compounds identified in that propolis were hexadecanoic acid, octadecanoic acid methyl ether, and 1-triacontanol methyl ester. The metabolites were identified using mass spectrometry [22].

3-O-Acetyl-α-amyrin (**2a**), 3-O-acetyl-β-amyrin (**2b**), lupeol (**3**), α-amyrin (**4a**), and β-amyrin (**4b**) are pentacyclic triterpenes ubiquitously distributed throughout the plant kingdom, in an accessible form, as aglycones, or in combined forms. They have long been known for their biological effects. α- and β-amyrin are commonly found in medicinal plants, and oleoresin is obtained by bark incision from Bursera or Protium of the Burseraceae family [18].

Lupeol (**3**) is found in many vegetal sources, such as mango, strawberry, cabbage, pepper, and the medicinal plant licorice. Extensive studies have proven that this compound has anti-inflammatory, antioxidant, anticancer, antimicrobial, and other pharmacological effects [23]. Lupeol protects the heart, liver, and skin [24] in some specific disease models.

Cicloartenol-23-en-3β,25-diol (**5**) is a triterpene produced by Lippia mexicana, which grows in the Mexican states of Oaxaca and Veracruz. Traditionally, this plant is used to treat coughs and gastrointestinal disorders and is also recommended for treating diabetes, liver diseases, and hypertension [19]. Furthermore, cicloartenol-23-en-3β,25-diol has exhibited 80% anti-inflammatory activity at a concentration of 100 mM [25]. Sachin et al. demonstrated its antibacterial activity against Bacillus subtilis, Staphylococcus aureus, and Escherichia coli and antifungal activity against Candida albicans at a concentration of 100 µL/mL. Additionally, an antidiabetic effect of cicloartenol-23-en-3β,25-diol was reported in mice. The animals showed a reduction in glucose levels; the suspected mechanism of action was related to an increase in pancreatic insulin secretion [26].

Alkylresorcinols are natural compounds found in higher plants. They possess a lipophilic polyphenol structure and show numerous biological properties, including antimicrobial, anticancer, antilipidemic, and antioxidant activities [21,27].

In a previous study, a bioassay-directed extraction and purification of mango peels allowed the isolation of 5-(11’Z-heptadecenyl)-resorcinol and 5-(8’Z,11’Z-heptadecadienyl)-resorcinol. Both compounds exhibited potent cyclooxygenase (COX-1 and COX-2) inhibitory activity with IC_50_ values ranging from 1.9 to 3.5 μM and 3.5 to 4.4 μM, respectively [21].

In addition to the identification of the main compounds in the propolis samples, we carried out a headspace solid-phase microextraction with gas chromatography and mass spectrometry time-of-flight (HS-SPME-GC-TOF-MS) analysis to identify the volatile compounds. The DVB/CAR/PDMS fiber used in this study has been employed to extract volatile components from previous work using geopropolis collected by our group in Coatepec [28]. As shown in Table S1, 39 volatile compounds were identified from the DB-5 column. At first, a tentative identification of the selected compounds was performed using NIST libraries [17]. The main volatile compounds identified were trans-α-bergamotene (8.15%), 2-heptanone (7.60%), hexanal (7.17%), α-copaene (7.09%), caryophyllene (5.67%), caryophyllene oxide (4.89%), and (2)-aromadendren oxide (4.08%), Table S1. It is interesting to note that this differs from a prior study of a sample of geopropolis produced by Melipona beecheii collected in Coatepec, Veracruz, Mexico. The main compounds detected included β-fenchene (14.53–15.45%), styrene (8.72–9.98%), benzaldehyde (7.44–7.82%), and terpenoids (58.17%) as the most relevant volatile components [28].

### 2.3. Total Polyphenol and Flavonoid Content of EEP

The total polyphenolic compounds for the recollected samples ranged from 2.45 ± 0.03 mg GAE/g to 3.48 ± 0.56 mg GAE/g of dry extract and flavonoid contents from 0.69 ± 0.03 mg QE/g to 0.84 ± 0.009 mg QE/g of dry extract (Table 1). These findings are similar to those observed for the ethanolic extract of propolis reported by Regnier et al. [29] with a total phenolic content of 0.62 ± 0.02 and 1.08 ± 0.14 mg GAE/g for *Melipona marginata* (“manduri”) and *Scaptotrigona xanthotricha* (“mandaguari amarela”). In contrast, in the work carried out by Ferreira et al. [30], the evaluation of the total phenolic and flavonoid content of the ethanol extract of propolis produced by *Scaptotrigona postica* (“mandaguari”) in the state of Rio Grande do Norte showed values of 111.5 ± 5.4 mg GAE/g of dry extract and 98.5 ± 8.6 mg QE/g, respectively. Gerginova et al. [14], in a previous study using samples of *Scaptotrigona. mexicana* propolis collected in two different locations of Chiapas, Mexico, demonstrated that the climate and specific flora around the hives influence the chemical composition of propolis collected by bees of the same species.

The phenolic and flavonoid content in propolis is a quality parameter of the material [31]. The Official Mexican Standard for Propolis, NOM-0003-SAG/GAN-2017 [32], sets the specifications for production, physical and chemical characteristics, and antimicrobial properties that propolis and its extracts must meet for processing and commercialization in the country. The values obtained for the extract are lower than those set by the Mexican Standard (minimum amount for phenols 50 mg GAE/g, and for flavonoids 5 mg QE/g).

Regarding antioxidant activity, the EEP was evaluated for its ability to quench the DPPH. The IC_50_ values ranged from activity 287.6 ± 7.8 μg/mL to 397.1 ± 9.3 μg/mL, higher than ascorbic acid (IC_50_ = 39.3 ± 2.9 μg/mL) used as a positive reference and Trolox (IC_50_ = 9.7 ± 3.4 μg/mL) (Table 1).

In the ABTS assay, the antioxidant activity is measured as the ability of test compounds to decrease the color by reacting directly with the radical ABTS^•+^ [33]. The EEP showed weak activity with IC_50_ values ranging from 438.7 ± 8.5 to 496.1 ± 6.8 (Table 1). These results are in agreement with those reported by Gerginova et al. [14] in a previous study conducted with samples collected from two distinct locations in Chiapas, Mexico. The propolis sample does not meet the quality parameters of Mexican Standard NOM-0003-SAG/GAN-2017, having a minimum inhibitory concentration of 160.7 µg/mL higher than established (IC_50_ < 100 µg/mL).

In the antiradical activity (FRAP), IC_50_ values ranged between 280.6 ± 9.3 to 301.6 ± 7.9 μg/mL for the extract. These results were in line with the ones obtained by Vongsak et al. [34] for propolis extracts produced by stingless bees *Lepidotrigona ventralis* Smith, *L. terminate* Smith, and *Tetragonula pagdeni* Schwarz in Thailand.

### 2.4. Antibacterial Activity of EEP

The antibacterial activity of the stingless bee propolis was determined based on the microdilution method in a 96-well plate using four medically relevant bacteria (Table 2). The ethanolic extract showed a slight inhibitory activity on *Staphylococcus aureus* and *Streptococcus mutans* growth at 512 and 256 µg/mL concentrations, respectively. At the same time, no inhibition was observed for *P. aeruginosa* and *P. gingivalis* at concentrations below 512 µg/mL. Stingless bee propolis is known for its medicinal properties, including its antiseptic and antibacterial properties [8]. This activity is related to its chemical composition, which varies according to the local vegetation, season, and bee species that generate this product [11]. In this study, the extract showed antibacterial activity against *Streptococcus mutans* (256 μg/mL) and *Staphylococcus aureus* (512 μg/mL).

The minimum inhibitory concentration values exhibited by the extracts (>512 µg/mL) for Gram-negative bacteria (*Pseudomonas aerugunosa* and *Phorphyromonas gingivalis*) were higher than those of Gram-positive bacteria. Przybyłek and Karpinski [35], in a previous study, reported that propolis exhibits antibacterial activity by increasing the permeability of the cell membrane, disrupting membrane potential and adenosine triphosphate production, and decreasing bacterial motility. These antibacterial mechanisms of propolis are closely linked to its chemical composition, particularly the varying proportions of terpenes and phenolic compounds. Lipophilic compounds, such as terpenes, are well documented in the literature for their antimicrobial properties [36]. Other authors emphasized that the antimicrobial activity of different propolis samples is related to the presence of volatile terpenes such as γ-elemene, α-ylangene, and valencene. These compounds can penetrate cell membranes, leading to the loss of essential intracellular components and ultimately causing microbial cell death [37]. The samples collected in the Totononacapan region of Mexico were rich in terpenes and volatile compounds with the presence of taraxeryl acetate (**1**), lupeol (**3**), and the mixtures of 3-*O*-acetyl-α-(**2a**) and 3-*O*-acetyl-β-amyrins (**2b**), and α-(**4a**) and β-amyrins (**4b**). The main volatile compounds detected include the terpenes *trans*-α-bergamotene, α-copaene, and the aldehyde hexanal.

## 3. Materials and Methods

### 3.1. Chemicals and Reagents

6-Hydroxy-2,5,7,8-tetramethylchroman-2-carboxylic acid (Trolox, 97%), gallic acid, 2,2-diphenyl-1-picrylhydrazyl (DPPH), Folin-Ciolcateau reagent 2N, aluminum chloride, sodium carbonate, sanguinarine, chlorhexidine digluconate, chloramphenicol, quercetin, ethanol, hexanes, ethyl acetate, dichloromethane, ferric chloride, cysteine hydrochloride, menadione, hemin, potassium nitrate, tetramethylsilane, ethyl acetate, hexanes, acetone, dichloromethane, and deuterated chloroform (CDCl_3_) were obtained from Sigma-Aldrich (St. Louis, MO, USA). Brain heart infusion broth was purchased from Becton Dickinson (BD, Franklin Lakes, NJ, USA). Gel 60 (0.063–0.200 mm) for column chromatography (70–230 mesh ASTM) was purchased from Merck-Millipore (Merck KGaA, Darmstadt, Germany).

### 3.2. Stingless Bee Propolis Sample

Finca la Isla Sociedad de Produccion Rural de Responsabilidad Limitada from Veracruz, Mexico, kindly provided raw stingless bee propolis samples from a meliponary located in Papantla, Mexico (20.362503 N, −97.243106 W). Four samples were harvested from technified wooden boxes in aseptic conditions in 2020, frozen, crushed, and stored at −20 °C until use.

### 3.3. Instrumentation

Nuclear magnetic resonance (NMR) spectra were registered using a Bruker AVANCE III 400 (400 MHz) and Bruker AVANCE III HD 700 (700 MHz) spectrometer, with tetramethylsilane (TMS) as the internal standard.

Crighton et al. described the conditions for the Direct Sample Analysis-Time of Flight (DSA-TOF) experiments (Perkin-Elmer, Waltham, MA, USA) [38].

Gas Chromatography-Mass Spectrometry GC-MS analyses were performed following the experimental conditions described by Rivero et al. [39]. Briefly, a gas chromatograph (6890 N series, Agilent Technology, Palo Alto, CA, USA) was coupled to a TOF mass spectrometer (LECO Corporation, St. Joseph, MI, USA) and fitted with a 5% diphenyl–95% dimethyl polysiloxane (20 m × 0.18 mm i.d.; 0.18 μm film thickness) capillary column (Bellefonte, PA, USA) for the analyses.

### 3.4. Extraction and Isolation of Compounds ***1***–***6*** from PEE

The air-dried and powdered stingless bee propolis was processed as described in Rivero-Cruz et al. [39]. The resin was extracted with ethanol for up to two weeks, and the extract was concentrated in vacuo. 60 g of the extract were subjected to a vacuum column chromatography (VLC) with silica gel and eluted with a solvent gradient mixture of dichloromethane-acetone (1:0 to 0:1) to give seven pooled fractions (F1–F7). A white solid spontaneously precipitated from fraction F1 (2.9 g) and was recrystallized from methanol, yielding 58.2 mg of **1** as white crystals. The remanent of F1 was subjected to column chromatography using silica gel impregnated with 10% silver nitrate as the support. Elution was performed with a gradient mixture of hexane: ethyl acetate (1:0 to 0:1). Compounds **2a** and **2b** were then purified as a mixture using preparative thin-layer chromatography with hexane: ethyl acetate: dichloromethane (8:1:1). Fraction F2, eluted with hexane: ethyl acetate (9:1), was chromatographed on a silica gel column, using a gradient mixture of hexane: ethyl acetate (1:0 to 0:1) as eluent, to give six fractions. Fraction F2-3 (100 mg) was separated by TLC with hexane: dichloromethane: acetone (8:1:1) to provide **3** (20.0 mg). Fraction F2-3 was chromatographed on an open column using silica gel and hexane mixtures: ethyl acetate (1:0 to 0:1) to give six subfractions (F2-3-1–F2-3-6). Subfraction F2-3-2 yielded crystals of compounds **4a** and **4b**. F2-3-4 (250.3 mg) yielded crystals of **5** (29.0 mg). Subfraction F2-3-6 (980 mg) yielded crystals of **6** (25.0 mg). Fraction F4 was chromatographed on silica gel using chloroform: acetone gradient (1:0 to 0:1) to give 10 fractions (F4-1–F4-10). Subfraction F4-6 was identified as **7**.

### 3.5. Headspace Solid-Phase Microextraction (HS-SPME), GC-MS-TOF, and Identification of Volatile Components

The volatile compounds of the stingless bee propolis were identified following the methodology previously described by Rivero et al. [16]. Briefly, 500 mg of propolis, 100 mg sodium chloride, and 10 mL of distilled water were mixed in a 25 mL vial with a rubber cap, and a Stableflex^®^ fiber 50/30 mm DVB/CAR/PDMS (1 cm) was inserted and exposed to the headspace for 15 min. Afterward, the fiber was retracted into the device, inserted into the GC injector port, and thermally desorbed. The desorption time was 2 min at 250 °C. The relative proportion of each component adsorbed to the fiber was calculated based on the analytical ion chromatogram (AIC) peak areas as a percentage of the sum of all peak areas.

The volatile constituents of stingless bee propolis were identified following the reported methodology [39]. The Kovats indices were calculated in relation to the retention times of a series of alkanes (C-8–C-20) compared to those of the chemical compounds gathered by Adams [40] and by comparing their MS fragmentation patterns with those of compounds in the spectral database of the National Institute of Standards and Technology (NIST) [41].

### 3.6. Physicochemical Characterization of the Stingless Bee Propolis Extract

#### 3.6.1. Antioxidant Activity

The DPPH radical scavenging capacity was used to determine the antioxidant activity of the samples, following the methodology described by Kahraman et al. [42] with some modifications. The determination was performed in 96-well plates. 100 µL of sample solution (1 mg/mL) was added, followed by 100 µL of ethanolic DPPH solution (0.208 mM). The samples were incubated at room temperature for 20 min, and absorbance was measured at 490 nm using a plate reader. The percentage inhibition of the DPPH of the samples was calculated considering the percentage of the steady DPPH in solution after the reaction [% inhibition = 100 (A_control_ − A_sample_)/A_control_]. All the determinations were performed in triplicates. The IC_50_ values were calculated from the relationship curve of scavenging activities (%) versus concentrations of the respective sample curve.

The ABTS test was performed according to a reported methodology by Re et al. [43], slightly modified. The ABTS + radical was formed by the oxidation reaction of a 7 mM ABTS solution with a 2.45 mM potassium persulfate solution. The resulting solution was stored overnight without light before use. Antioxidant activity was evaluated by the capacity of the samples to scavenge the ABTS + radical. The ABTS + radical solution was diluted with MeOH to achieve an initial absorbance of at least 0.70 at 700 nm. The assay was carried out in 96-well plates. 20 µL of each sample solution (1 mg/mL) was added, along with 180 µL of the ABTS + radical solution, adjusted to an absorbance of at least 0.70. The samples were incubated at room temperature for 6 min, and absorbance was measured at 700 nm using a plate reader.

The iron-reducing capacity determination (FRAP) assay was performed using the methodology described by Benzie et al. [44] with some modifications. The assay was carried out in 96-well plates, with each well containing 20 µL of sample solution (0.1 mg/mL) and 180 µL of FRAP reagent solution (prepared with 2.5 mL of 10 mM 2,4,6-tripyridyl-S-triazine solution in 40 mM HCl, 2.5 mL of 20 mM FeCl_3_, and 25 mL of 300 mM acetate buffer at pH 3.6). The samples were incubated at room temperature for 30 min, and absorbance was measured at 595 nm using a plate reader.

#### 3.6.2. Phenolic and Flavonoid Content

The total phenolic content of the propolis was determined as described by Singleton and Rossi [45]. All the determinations were performed in triplicates. The obtained absorbances were interpolated into a calibration curve of gallic acid. The results were expressed as mg equivalents of gallic acid/g of dry propolis extract (EEP).

The concentration of flavonoids was achieved using the method described by Marquele et al. [46] using the aluminum chloride reagent. The obtained absorbances were interpolated in a calibration curve of quercetin. The results were expressed as mg equivalents of quercetin/g of dry propolis extract (EEP).

### 3.7. Bacterial Strains for the Antimicrobial Activity Tests

The antibacterial activity was tested using two Gram-positive strains, *Streptococcus mutans* (ATCC 10449) and *Staphylococcus aureus* (ATCC 25923), and two Gram-negative strains, *Porphyromonas gingivalis* (ATCC 33277) and *Pseudomonas aeruginosa* (ATCC 27853). Bacteria were grown in brain heart infusion broth (BHI), except for *Porphyromonas gingivalis*, which was cultured in trypticase soy broth-yeast extract medium supplemented with cysteine hydrochloride (0.05%), menadione (0.02 g/mL), hemin (5 g/mL), and potassium nitrate (0.02%). The bacterial inocula were prepared by suspending microbial growth in BHI broth adjusted to a turbidity equivalent to 0.5 on the McFarland scale (10^7^–10^8^ CFU/mL) at 620 nm using a spectrometer. All the strains were incubated at 37 °C for 24 h; *Porphyromonas gingivalis* was under anaerobic conditions in GasPak jars, while the other strains were under aerobic conditions.

### 3.8. Minimum Inhibitory Concentration of the Stingless Bee Propolis Extract

The in vitro antibacterial activity of the EEP was determined according to the Clinical and Laboratory Standards Institute (CLSI), which recommended minimum inhibitory concentration (MIC) protocol with slight modifications [47]. Briefly, twofold dilutions were made for all the tested antibacterial agents starting from 1000 μg/mL to 3.9 μg/mL in a 96-well plate. An aliquot of 20 μL of bacterial suspension was added to 180 μL of antibacterial dilution. Each well in the microtiter plate contained *Streptococcus mutans* [final concentration of 5 × 10^5^ colony forming units (CFU)/mL] or *Porphyromonas gingivalis* (5 × 10^6^ CFU/mL). The EEP was dissolved in dimethylsulfoxide to a final concentration of 5% (*v*/*v*), and this solution was used as a negative control. Chlorhexidine gluconate, chloramphenicol, and sanguinarine were used as positive controls. BHI was used as a negative control and as a sterile control. After the incubation period, the resazurin method was carried out for the MIC determination, using a 0.01% (*w*/*v*) sodium resazurin ethanolic solution, following the described methodology [48,49]. Briefly, 10 μL of the resazurin solution was applied to each well for visual interpretation; blue indicated bacterial inactivity, and pink indicated bacterial growth. In the presence of resazurin, the test shows blue [49].

## 4. Conclusions

Mexico, a megadiverse country, produces various types of propolis from stingless bees, which are traditionally used to treat numerous illnesses. In this study, we isolated nine known components (**1**–**7**) and identified 39 volatile compounds from Mexican stingless bee propolis collected in Veracruz, Mexico. Our findings provide evidence that supports the biological properties of *Scaptotrigona mexicana* propolis. The chemical study of stingless bee propolis from Mexico and its pharmacological activities remain largely unexplored. Further research is needed to confirm the pharmacological properties of stingless propolis. Furthermore, it is worth noting that additional studies are underway to enhance the extraction process of *Scaptotrigona mexicana* geopropolis using alternative solvents, and the results will be presented in due course. Ultimately, the screening of other biological activities is recommended.

## Figures and Tables

**Table 1:** Total phenolic content (TPC), total flavonoid content (TFC), and antioxidant/antiradical activities of propolis extract.

EEP Sample	Total Phenolics mg GAE/g ^a^	Total Flavonoids mg QE/g ^a^	DPPH Scavenging IC_50_ μg/mL ^a^	ABTS IC_50_ μg/mL ^a^	FRAP IC_50_ μg/mL ^a^
EEP-1	2.45 ± 0.03	0.69 ± 0.03	368.9 ± 12.6	438.7 ± 8.5	280.6 ± 9.3
EEP-2	3.47 ± 0.22	0.78 ± 0.006	287.6 ± 7.8	489.7 ± 5.2	286.4 ± 5.2
EEP-4	3.48 ± 0.56	0.84 ± 0.009	397.1 ± 9.3	496.1 ± 6.8	301.6 ± 7.9
EEP-5	2.68 ± 0.22	0.82 ± 0.002	303.6 ± 6.7	454.4 ± 7.5	294.5 ± 3.7
Trolox	-	-	9.7 ± 3.4	13.9 ± 0.62	

^a^ Values are expressed as means ± standard error, *n* = 3.

**Table 2:** Antimicrobial activity of stingless bee ethanolic extract (PEE) and positive controls.

Compounds	MIC (μg/mL)			
	*S. mutans*	*S. aureus*	*P. aeruginosa*	*P. gingivalis*
Propolis Ethanolic extract	256	512	>1024	512
Sanguinarine ^a^	12.5	50	250	125
CHX ^a,b^	1.0	2.0	200	12.0
Chloramphenicol ^a^	2.0	12.5	600	12.0

^a^ positive control; ^b^ Chlorhexidine digluconate.

## Data Availability

The data presented in this study are available upon request from the corresponding author.

## Supplementary Materials

The following supporting information can be downloaded at https://www.mdpi.com/article/10.3390/molecules30061370/s1, Table S1. Volatile components from stingless bee propolis; Figure S1. ^1^H NMR (400 MHz, CDCl_3_) of taraxeryl acetate (1); Figure S2. ^1^H NMR (700 MHz, CDCl_3_) of the mixture of 3-*O*-acethyl-α-amyrin (2a) and 3-*O*-acethyl-b-amyrin (2b); Figure S3. ^1^H NMR (400 MHz, CDCl_3_) of lupeol (3); Figure S4. ^1^H NMR (400 MHz, CDCl_3_) of of the mixture of α and β-amyrins (4a and 4b); Figure S5. ^1^H NMR (400 MHz, CDCl_3_) of cycloart-23-en-3β,25-diol (5); Figure S6. ^1^H NMR (400 MHz, CDCl_3_) of mangiferoic acid (6); Figure S7. ^1^H NMR (400 MHz, CDCl_3_) 5-(11’*Z*-heptadecenyl)-resorcinol (7).
