# Supplementary material for: Scaptotrigona mexicana Propolis from Totonacapan Region: Chemical Composition, Antioxidant and Antibacterial Activities
Source: Molecules. 2025 Mar 19;30(6):1370. doi: 10.3390/molecules30061370 (PMC11944469; doi:10.3390/molecules30061370)
Supplement: Supplementary file 1 [file molecules-30-01370-s001.zip › molecules-3499344-supplementary.pdf]

molecules

MDPI

Suplementary material

Scaptotrigona mexicana proplis from Totonacapan region: Chemical composition, antioxidant and antibacterial activities.

Blanca E. Rivero-Cruz,[1,*] Maria Ema Rojas-Brandao,[1] Adriana Correa-Benítez,[2] Ingeborg Becker,[3] Aurora Xolalpa-Aroche,[4] José Delgado-Dominguez,[3] J. Fausto Rivero-Cruz,[4,5*]

[1] Facultad de Química, Universidad Nacional Autónoma de México, Ciudad Universitaria, Ciudad de Mexico 04510, Mexico; 317516760@quimica.unam.mx
[2] Facultad de Medicina Veterinaria y Zootecnia, Universidad Nacional Autónoma de Mexico, Ciudad Universitaria, Ciudad de Mexico 04510, Mexico; adrianac@unam.mx
[3] Facultad de Médicina, Universidad Nacional Autónoma de Mexico, Ciudad Universitaria, Ciudad de Mexico 04510, Mexico; becker@unam.mx (I.B.); josesoterod@gmail.com (J.D.-D.)
[4] Centro de Innovacion para el Desarrollo Apícola Sustentable en Quintana Roo, Universidad Intercultural Maya de Quintana Roo, Jose Maria Morelos 77890, Mexico; aurora.xolalpa@uimqroo.edu.mx
[5] Investigadores por Mexico, Comisión Intersecretarial de Bioseguridad de los Organismos Genéticamente Modificados (CIBIOGEM-SECIHTI), Ciudad de Mexico 03940, Mexico
* Correspondence: blancariv@unam.mx (B.E.R.-C.); jose.rivero@secihti.mx (J.F.R.-C.)

List of content:

Table S1. Volatile components from stingless bee propolis
Figure S1. ^{1}H NMR (400 MHz, $CDCl_3$) of taraxeryl acetate (**1**)
Figure S2. ^{1}H NMR (700 MHz, $CDCl_3$) of the mixture of 3-*O*-acethyl-α-amyrin (**2a**) and 3-*O*-acethyl-β-amyrin (**2b**)
Figure S3. ^{1}H NMR (400 MHz, $CDCl_3$) of lupeol **(3)**
Figure S4. ^{1}H NMR (400 MHz, $CDCl_3$) of of the mixture of α and β-amyrins (**4a** and **4b**)
Figure S5. ^{1}H NMR (400 MHz, $CDCl_3$) of cycloart-23-en-3β,25-diol (**5**)
Figure S6. ^{1}H NMR (400 MHz, $CDCl_3$) of mangiferoic acid (**6**)
Figure S7. ^{1}H NMR (400 MHz, $CDCl_3$) 5-(11´*Z*-heptadecenyl)-resorcinol (**7**);

Table S1. Volatile components from stingless bee propolis.

Compound	Molecular formula	Kovats Index	Area (%)
Hexanal[a, b, c]	$C_6H_{12}O$	801	7.172
2-heptanone[a, b, c]	$C_7H_{14}O$	892	7.598
5-methyl-2-hexanone[a, b]	$C_7H_{14}O$	899	1.891
α-pinene[a, b, c]	$C_{10}H_{16}$	939	0.979
(2*Z*)-heptenal[a, b]	C7H12O	954	2.141
Octanal[a, b]	$C_8H_{16}O$	998	0.786
o-cimene[a, b]	$C_{10}H_{14}$	1026	1.08
D-limonene[a, b]	$C_{10}H_{16}$	1029	2.144
1-octanol[a, b]	$C_8H_{18}O$	1068	0.95
Hexylpropionate[a, b]	$C_9H_{18}O_2$	1101	2.823
Octen-1-ol acetate[a, b, c]	$C_{10}H_{18}O_2$	1112	3.756
3-nonen-2-one[a, b]	$C_9H_{16}O$	1142	0.47
(2*Z*)-nonenal[a, b, c]	$C_9H_{16}O$	1149	1.272
Hexanoic acid penthyl ester[a, b, c]	$C_{11}H_{22}O_2$	1249	0.512
2-decen-1-ol[a, b]	$C_{10}H_{20}O$	1271	2.808
Pentanoic acid hexyl ester[a, b]	$C_{11}H_{22}O_2$	1299	0.514
(+)-cicloisosativene[a, b]	$C_{15}H_{24}$	1371	2.438
α-copaene[a, b]	$C_{15}H_{24}$	1376	7.094
Hexanoic acid hexyl ester[a, b, c]	$C_{12}H_{24}O_2$	1383	2.675
β-burbonene[a, b]	$C_{15}H_{24}$	1388	2.152
β-cubebene[a, b]	$C_{15}H_{24}$	1388	2.152
β-elemene[a, b]	$C_{15}H_{24}$	1390	1.687
α-gurjenene[a, b]	$C_{15}H_{24}$	1409	1.098
Caryophyllene[a, b, c]	$C_{15}H_{24}$	1419	5.671
trans-α-bergamotene[a, b]	$C_{15}H_{24}$	1434	8.145
Aromadendrene[a, b]	$C_{15}H_{24}$	1441	3.627
(*E*)-β-farnesene[a, b]	$C_{15}H_{24}$	1456	0.31
D-germacrene[a, b]	$C_{15}H_{24}$	1481	0.951
γ–selinene[a, b]	$C_{15}H_{24}$	1492	3.284
α-selinene[a, b]	$C_{15}H_{24}$	1498	0.976
α-muurolene[a, b]	$C_{15}H_{24}$	1500	3.284
cis-α-bisabolene[a, b]	$C_{15}H_{24}$	1507	0.544
trans-2-ethyl-2-hexen-1-ol[a, b]	$C_8H_{16}O$	1512	3.284
(+)-δ-cadinene[a, b]	$C_{15}H_{24}$	1523	1.109
Cadin-1,4-diene[a, b]	$C_{15}H_{24}$	1538	0.475

**Compound**	**Molecular formula**	**Kovats Index**	**Area (%)**
Caryophyllene oxide[a, b]	$C_{15}H_{24}O$	1583	4.849
(2)-aromadendrene oxide[a, b]	$C_{15}H_{24}O$	1584	4.089
α-santalol[a, b]	$C_{15}H_{24}O$	1675	1.867
Cadalene[a, b]	$C_{15}H_{18}$	1676	0.43
		**TOTAL**	**99.087**

Identification: (a) retention time; (b) retention index; (c) mass spectrum

Taraxeryl acetate (1): ^{1}H NMR (400 MHz, $CDCl_3$): δ_H 5.5 (dd, 1H, *J*= 8.4, 2.8 Hz, H-15), 4.47 (dd, 1H, *J*= 10.8, 5.1 Hz, H-3), 2.04 (s, 3H, C<u>H</u>$_3$-COO-), 1.09 (s, 3H, H-28), 0.95 (s, 6H, H-26 y H-27), 0.91 (s, 3H, H-30), 0.90 (s, 3H, H-29), 0.88 (s, 3H, H-24), 0.86 (s, 3H, H-23), 0.82 (s, 3H, H-25) (S2 Fig.). RMN^{13}C (100 MHz, $CDCl_3$): δ_C 171.14 (CH_3-<u>C</u>OO-), 158.14 (C-14), 117.09 (C-15), 81.17 (C-3), 55.79 (C-5), 49.10 (C-9), 48.55 (C-18), 41.26 (C-7), 39.14 (C-8), 38.05 (C-13), 37.84 (C-1), 37.71 (C-10), 37.64 (C-4), 37.44 (C-16), 36.79 (C-19), 35.91 (C-17), 35.25 (C-22), 33.83 (C-12), 33.50 (C-29), 33.08 (C-21), 29.96 (C-28), 29.08 (C-20), 27.98 (C-23), 25.98 (C-26), 23.62 (C-2), 21.47 (C-30), 21.44 (CO-C<u>H</u>$_3$), 21.07 (C-27), 18.84 (C-6), 17.66 (C-11), 16.74 (C-24), 15.65 (C-25) [1].

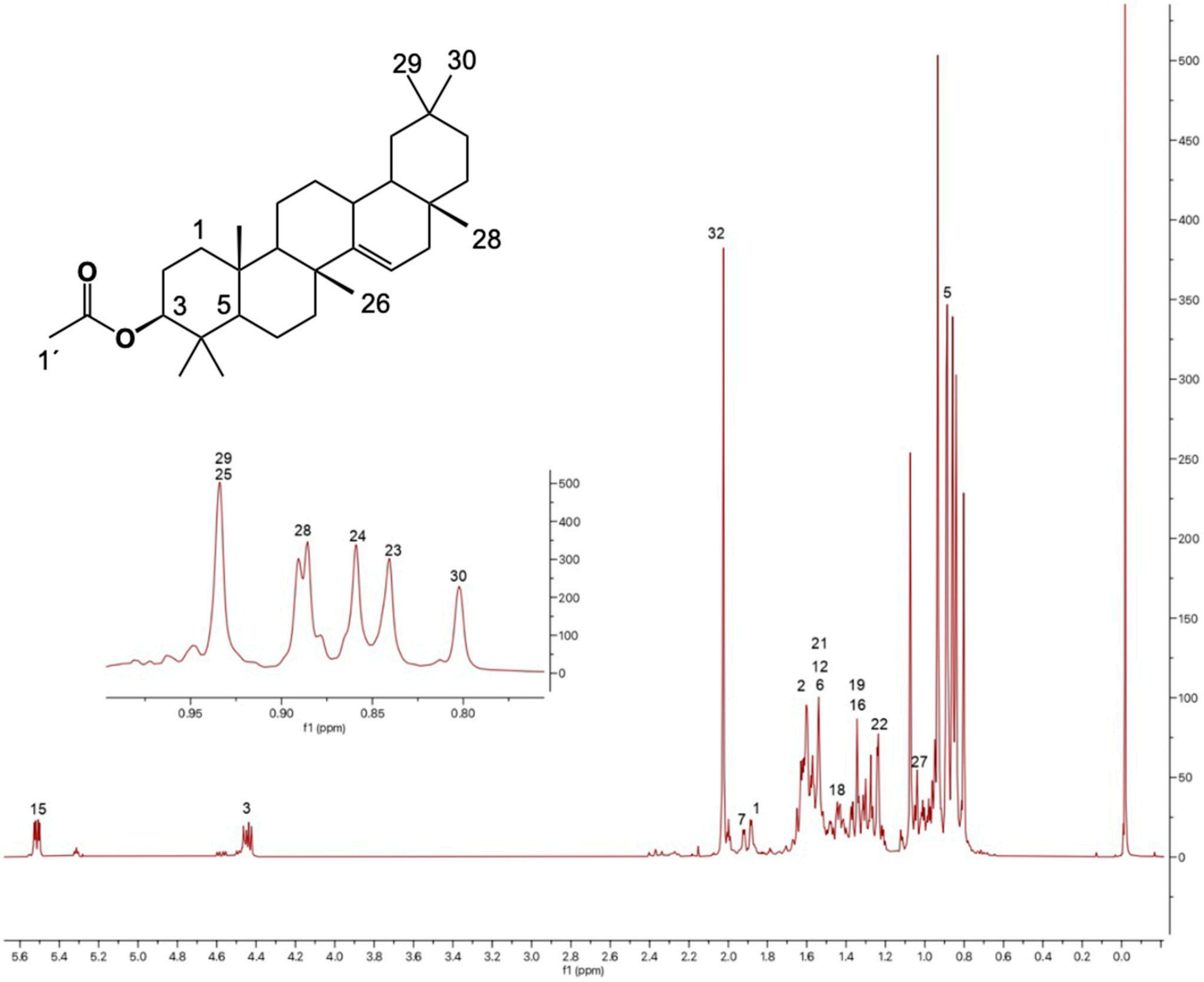

Figure S1. ^{1}H NMR (400 MHz, $CDCl_3$) of taraxeryl acetate (**1**)

3-*O*-Acetyl-α-amyrin (2a): ^{1}H NMR (700 MHz, $CDCl_3$): δ_H 5.12 (t, 1H, *J*= 3.6 Hz, 1H, H-12), 4.50 (m, 1H, H-3), 2.05 (s, 3H, H-1´), 1.33 (m, 1H, H-19), 1.06 (s, 3H, H-27), 1.01 (s, 3H, H-26), 0.98 (s, 3H, H-25), 0.92 (d, 3H, *J*= 6.2 Hz, H-30), 0.87 (s, 6H, H-23 y H-24), 0.80 (s, 3H, H-28), 0.79 (d, 3H, *J*= 6.2 Hz, H-29). ^{13}C NMR (176 MHz, $CDCl_3$): δ_C 171.17 (C-2´), 139.78 (C-13), 124.47 (C-12), 81.11 (C-3), 59.21 (C-18), 55.41 (C-5), 47.79 (C-9), 42.22 (C-14), 41.68 (C-22), 40.18 (C-8), 39.80 (C-19), 39.76 (C-20), 38.61 (C-1), 38.04 (C-4), 37.29 (C-10), 33.90 (C-17), 33.01 (C-7), 31.40 (C-21), 28.90 (C-28), 28.24 (C-16), 28.22 (C-23), 26.75 (C-15), 23.75 (C-2), 23.52 (C-11), 23.37 (C-27), 21.55 (C-30), 21.47 (C-1´), 18.39 (C-6), 17.66 (C-29), 17.01 (C-26), 16.89 (C-24), 15.89 (C-25). **3-*O*-acethyl-β-amyrin (2b):** ^{1}H NMR (700 MHz, $CDCl_3$): δ_H 5.18 (t, 1H, *J*= 3.6 Hz, H-12), 4.46 (m, 1H, H-3), 2.05 (s, 3H, H-1´), 1.67 (m, 2H, H-19), 1.13 (s, 3H, H-27), 0.97 (s, 3H, H-26), 0.96 (s, 3H, H-25), 0.87 (s, 3H, H-23), 0.86 (s, 9H, H-29, H-30, H-24), 0.83 (s, 3H, H-28). ^{13}C NMR (176 MHz, $CDCl_3$): δ_C 171.18 (C-2'), 145.37 (C-13), 121.79 (C-12), 81.10 (C-3), 55.40 (C-5), 47.70 (C-9), 46.93 (C-19), 41.86 (C-14), 39.96 (C-8), 38.41 (C-21), 37.97 (C-4), 37.86 (C-1), 36.99 (C-7), 36.94 (C-10), 34.88 (C-22), 33.48 (C-30), 32.73 (C-17), 31.24 (C-20), 28.55 (C-28), 28.22 (C-23), 27.07 (C-16), 26.28 (C-15), 26.10 (C-27), 23.84 (C-29), 23.72(C-2), 23.68 (C-11), 21.47 (C-1´), 18.41 (C-6), 16.95 (C-26), 16.89 (C-24), 15.71 (C-25) [2.].

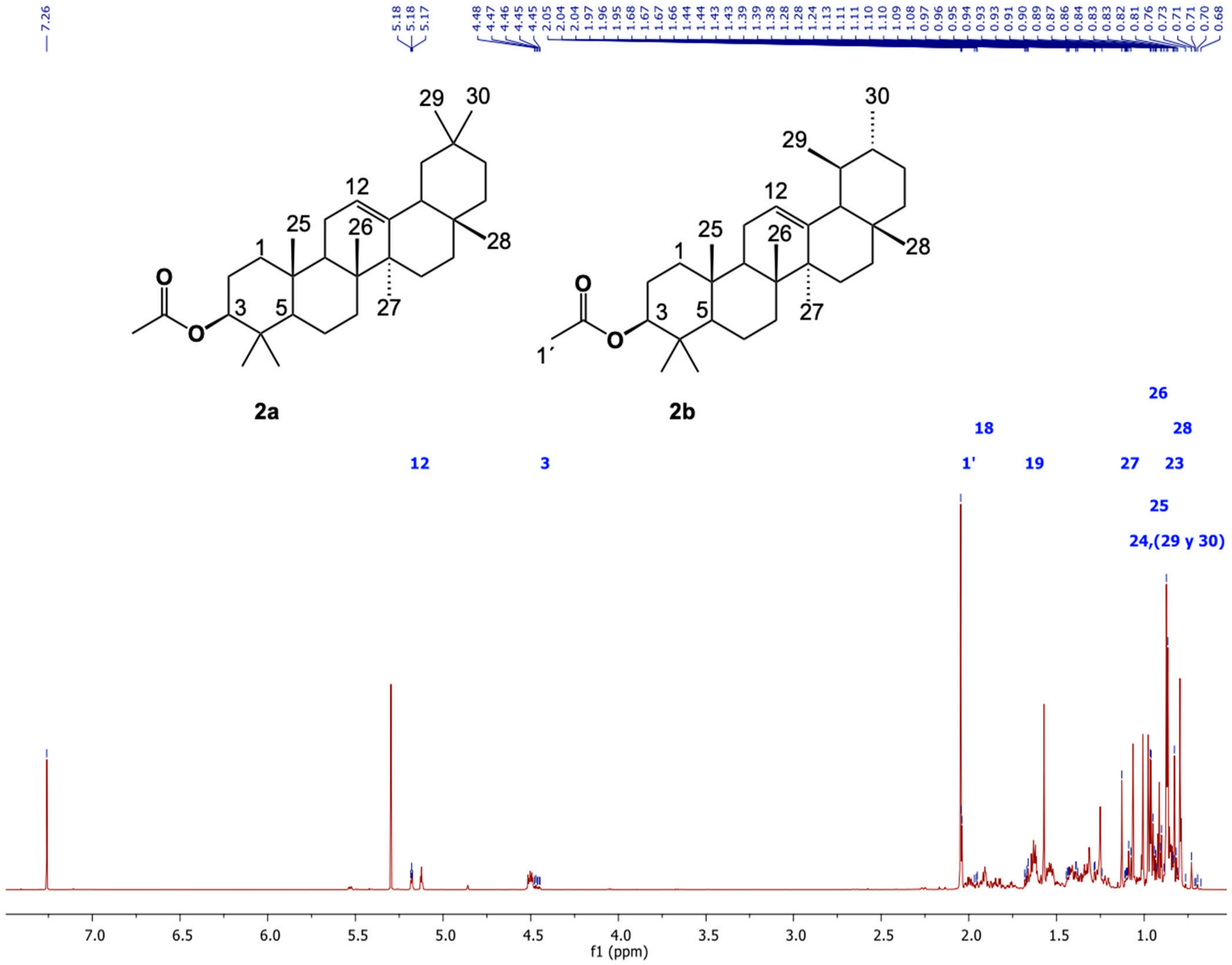

Figure S2. ^{1}H NMR (700 MHz, $CDCl_3$) of the mixture of 3-*O*-acethyl-α-amyrin (**2a**) and 3-*O*-acethyl-β-amyrin ( )

Lupeol (3): ^{1}H NMR (400 MHz, $CDCl_3$): δH 4.68 (d, 1H, *J* = 2.3 Hz, H-30a), 4.56 (dd, 1H, *J* = 2.4, 1.4 Hz, H-30b), 3.38 (t, 1H, *J* = 2.8 Hz, H-3), 2.37 (td, 1H, *J* = 11.1, 5.9 Hz, H-19), 1.67 (s, 3H, H-29), 1.01 (d, 3H, *J* = 9.2 Hz, H-25), 0.95 (s, 3H, H-27), 0.93 (s, 3H, H-23), 0.84 (s, 3H, H-26), 0.81 (s, 3H, H-24), 0.78 (s, 3H, H-28) (S4 Fig.). ^{13}C NMR (101 MHz, $CDCl_3$): δC 151.13 (C-20), 109.44 (C-30), 76.36 (C-3), 50.29 (C-9), 49.11 (C-5), 48.38 (C-18), 48.14 (C-19), 43.12 (C-17), 43.01 (C-14), 41.12 (C-8), 40.12 (C-22), 38.11 (C-13), 37.64 (C-4), 37.38 (C-10), 35.69 (C-16), 34.23 (C-1), 33.35 (C-7), 29.95 (C-21), 28.39 (C-23), 27.49 (C-15), 25.50 (C-2), 25.20 (C-12), 22.26 (C-24), 20.89 (C-11), 19.41 (C-29), 18.39 (C-6), 18.12 (C-28), 16.08 (C-25), 16.04 (C-26), 14.75 (C-27) [3].

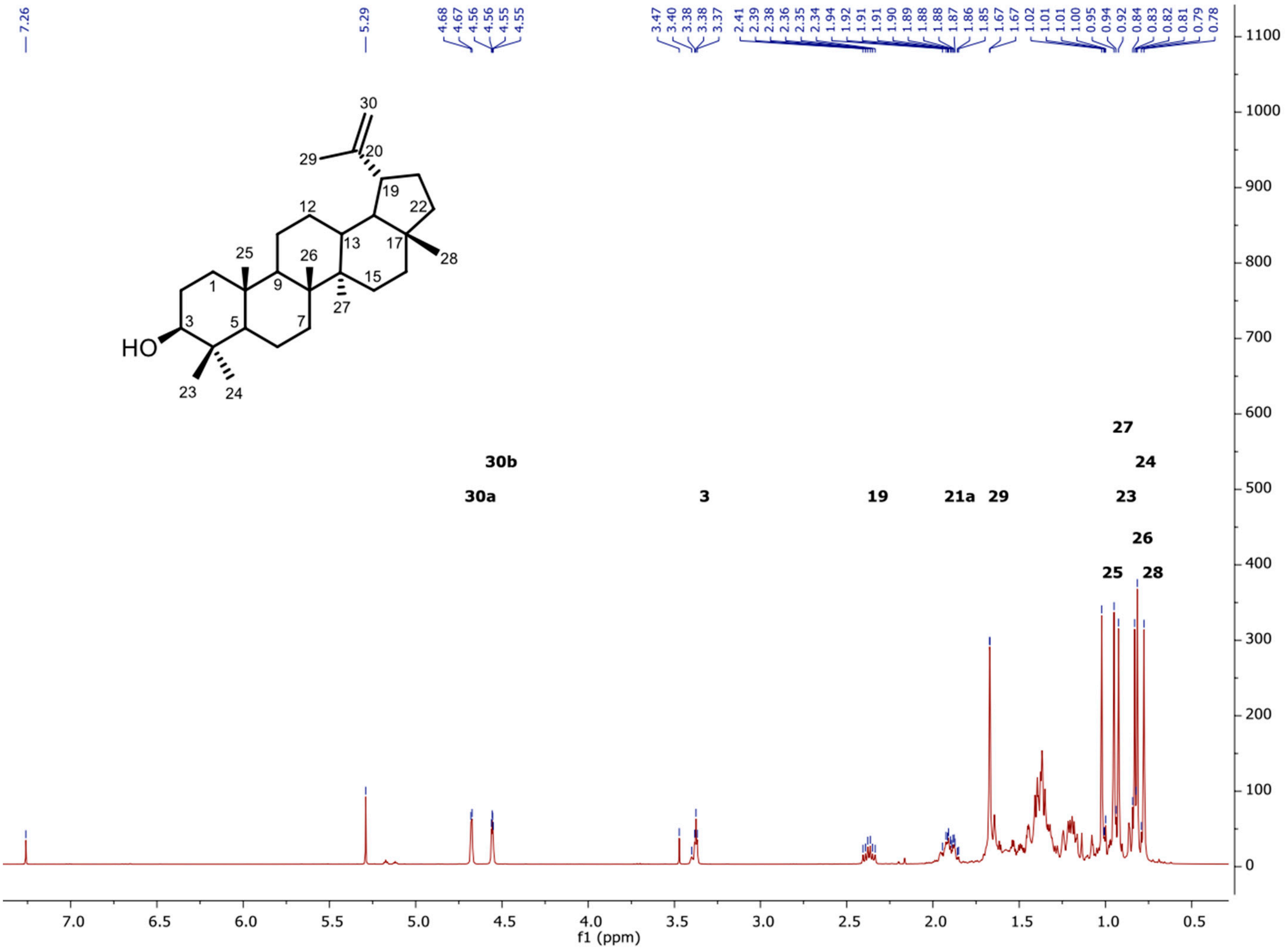

Figure 3. ^{1}H NMR (400 MHz, $CDCl_3$) of lupeol (**3**).

α and β-amyrins (C4a and C4b): ^{1}H NMR (400 MHz, $CDCl_3$): δ_H 5.13 (t, 1H, *J*= 3.6 Hz, H-12), 3.22 (dd, 1H, *J*= 10.8, 5.1 Hz, H-3), 1.07 (s, 3H, H-27), 1.01 (s, 3H, H-26), 1.00 (s, 3H, H-24), 0.95 (s, 3H, H-25), 0.92 (d, 3H, *J*= 5.1 Hz, H-30), 0.80 (s, 3H, H-28), 0.79 (s, 4H, H-23), 0.78 (s, 1H, H-29) (S5 Fig). ^{13}C NMR (100 MHz, $CDCl_3$): δ_C 139.71 (C-13), 124.53 (C-12), 79.19 (C-3), 59.17 (C-18), 55.29 (C-5), 47.83 (C-9), 42.19 (C-14), 41.65 (C-22), 40.13 (C-8), 39.78 (C-19), 39.74 (C-20), 38.90 (C-1, C-4), 37.02 (C-10), 33.89 (C-17), 33.05 (C-7), 31.39 (C-21), 28.89 (C-28), 28.26 (C-24), 28.22 (C-16), 27.39 (C-2), 26.74 (C-15), 23.49 (C-11), 23.40 (C-27), 21.56 (C-30), 18.48 (C-6), 17.62 (C-29), 16.99 (C-26), 15.82 (C-25), 15.77 (C-23) [3].

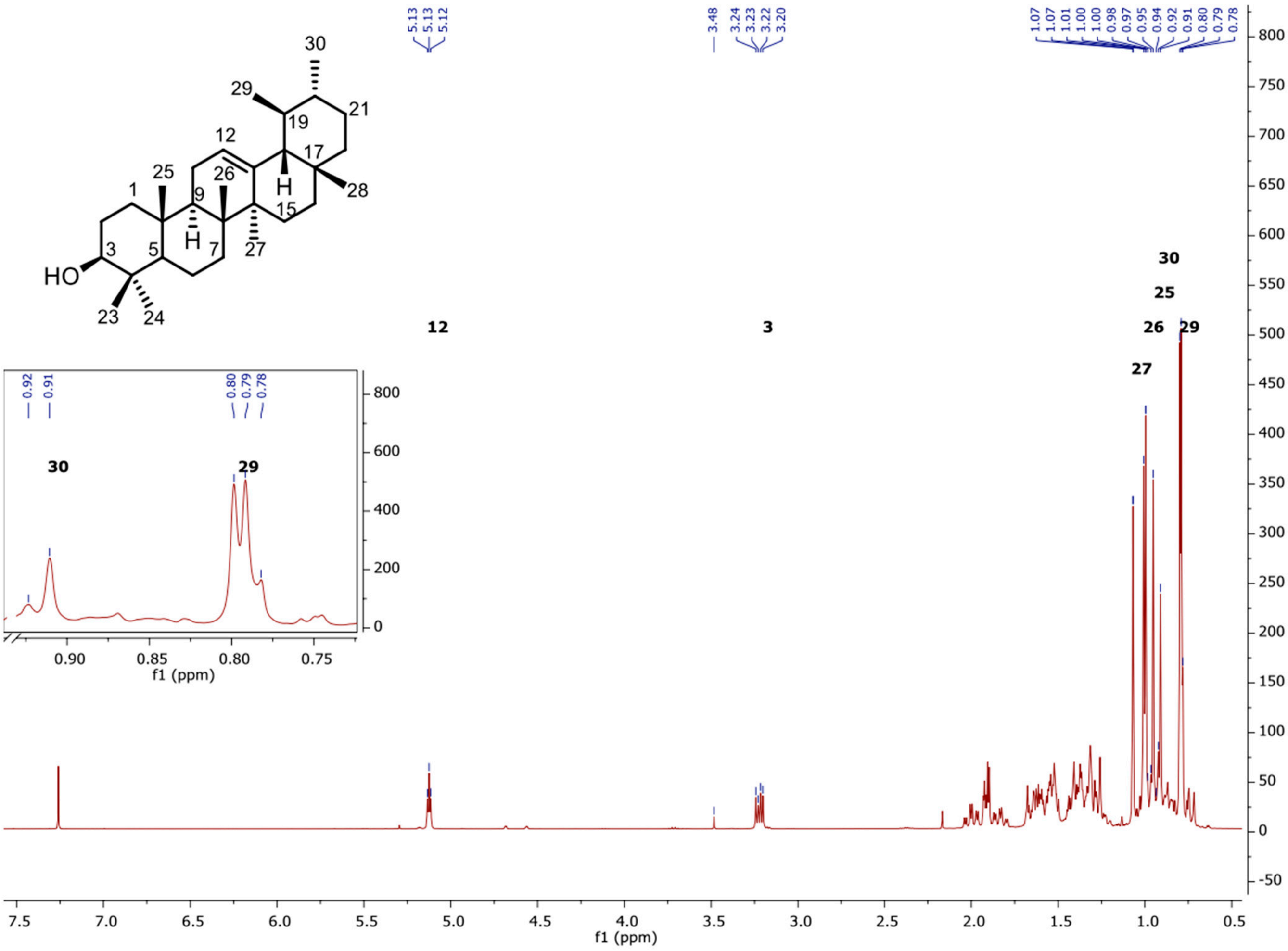

Figure 4. ^{1}H NMR (400 MHz, $CDCl_3$) of the mixture of α and β-amyrins (**C4a** and **C4b**).

Cicloart-23-en-3β,25-diol (5): ^{1}H NMR (400 MHz, $CDCl_3$): δ_H 5.59 (m, 2H, H-23 y H-24), 3.28 (dd, 1H, *J*= 11.2, 4.4 Hz, H-3), 1.31 (s, 6H, H-26 y H-27), 0.96 (s, 6H, H-29 y H-18), 0.88 (s, 3H, H-30), 0.86 (d, 3H, *J*= 6.4 Hz, H-21), 0.81 (s, 3H, H-28), 0.55 (d, 1H, *J*= 4.2 Hz, H-19a), 0.33 (d, 1H, *J*= 4.2 Hz, H-19b) (S6 Fig.). ^{13}C NMR (125 MHz, $CDCl_3$): δ_C 139.50 (C-24), 125.75 (C-23), 78.98 (C-3), 70.89 (C-25), 52.15 (C-17), 48.97 (C-14), 48.11 (C-8), 47.24 (C-5), 45.45 (C-13), 40.63 (C-4), 39.18 (C-22), 36.53 (C-20), 35.72 (C-15), 32.93 (C-1), 32.11 (C-12), 30.53 (C-2), 30.12 (C-19), 30.03 (C-25, C-26), 28.22 (C-7), 26.59 (C-16), 26.24 (C-10), 26.15 (C-11), 25.58 (C-29), 21.26 (C-6), 20.13 (C-9), 19.44 (C-30), 18.43 (C-21), 18.23 (C-18), 14.15 (C-28) [4].

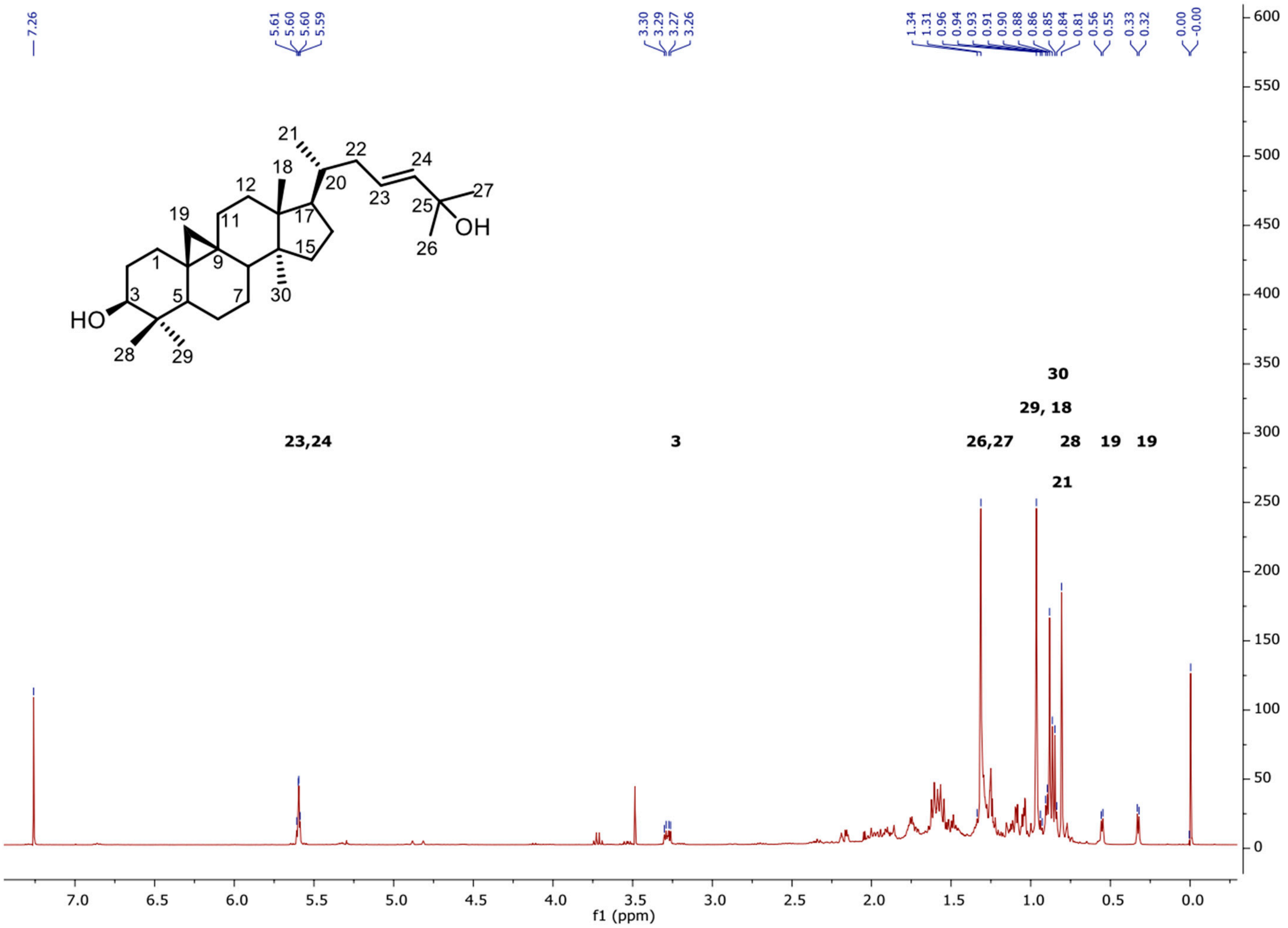

Figure 5. ^{1}H NMR (400 MHz, $CDCl_3$) of cycloart-23-en-3β,25-diol (**5**)

Mangiferoic acid (6): ^{1}H NMR (400 MHz, $CDCl_3$): δ_H 2.71 (td, 2H, *J*= 4.8 y 4.0 Hz, H-2), 1.86 (m, 1H, H-1a), 1.85 (s, 3H, H-27), 1.71 (d, 1H, *J*= 8.4, Hz, H-5), 1.10 (s, 3H, H-29), 1.05 (s, 3H, H-28), 1.00 (s, 3H, H-18), 0.91 (s, 3H, H-30), 0.79 (d, 1H, *J*=4.2, H-19a), 0.58 (d, 1H, H-19b) (S7 Fig.) ^{13}C NMR (100 MHz, $CDCl_3$): δ_C 216.84 (C-3), 172.85 (C-26), 145.95 (C-24), 126.69 (C-25), 52.35 (C-17), 50.39 (C-4), 48.89 (C-14), 48.57 (C-5), 48.02 (C-8), 45.53 (C-13), 37.63 (C-2), 36.10 (C-20), 35.68 (C-15), 34.91 (C-22), 33.57 (C-1), 32.93 (C-12), 29.70 (C-19), 28.29 (C-16), 26.84 (C-11), 26.11 (C-23), 26.05 (C-7), 26.01 (C-10), 22.32 (C-28), 21.64 (C-6) [5].

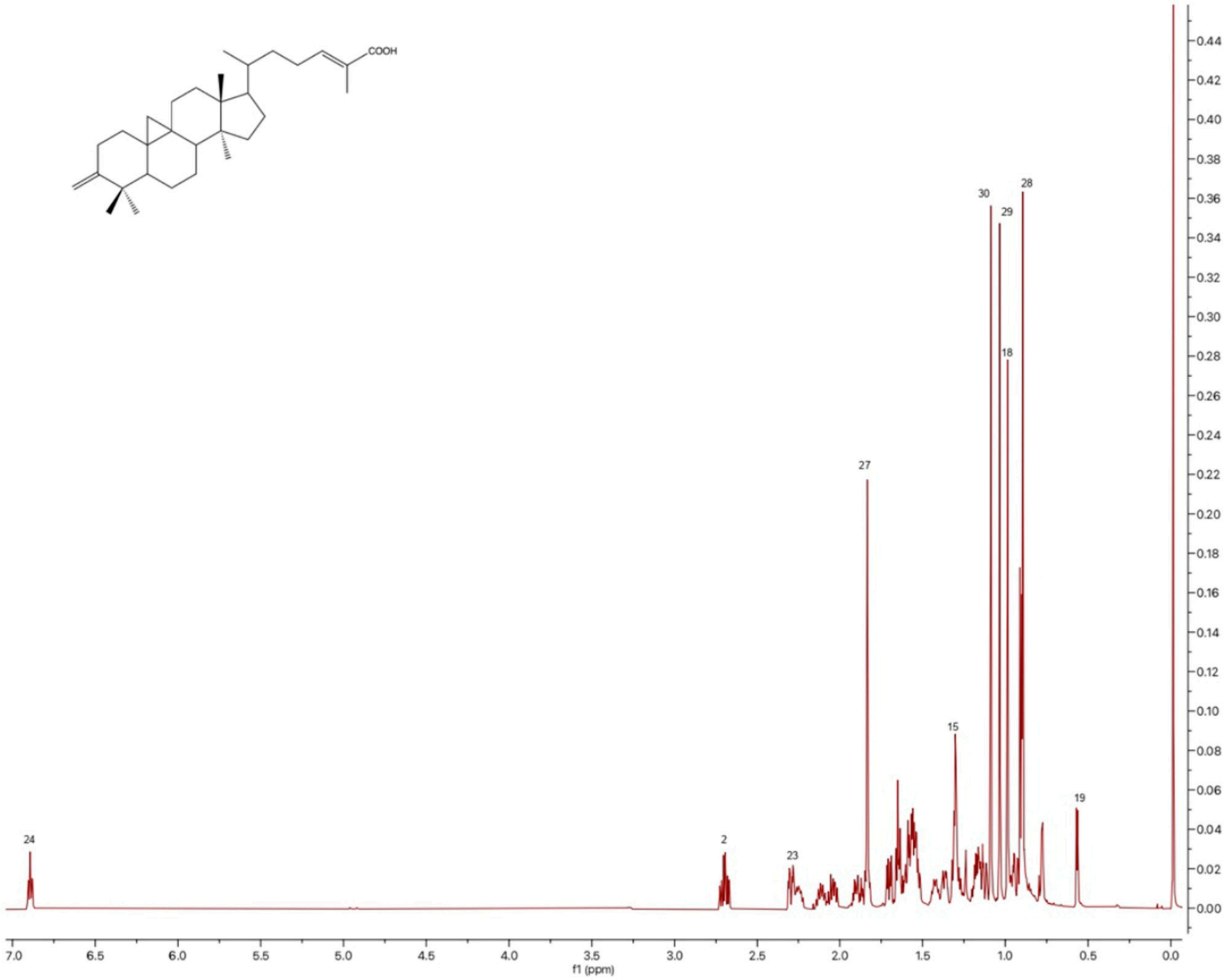

Figure 6. ^{1}H NMR (400 MHz, $CDCl_3$) of mangiferoic acid (**6**).

5-(11´*Z*-heptadecenyl)-resorcinol (7): ^{1}H NMR (400 MHz, $CDCl_3$): δ_H 6.24 (d, 2H, *J*= 1.0 Hz, H-6), 6.18 (s, 1H, *J*= 1.0 Hz, H-4), 5.35 (m, 2H, H-11' y H-12'), 2.48 (ta, 2H, J=8.0, 8.0, H-1'), 2.02 (m, 4H, H-10' y H-13'), 1.56 (m, 2H, H-2'), 1.32 (m, H-3' - H-9') (S8 Fig.). ^{13}C NMR (100 MHz, $CDCl_3$): δ_C 156.74 (C-1, C-3), 146.30 (C-5), 108.17 (C-4, C-6), 100.29 (C-2), 130.07 (C-11' y C-12'), 35.98 (C-1'), 32.12 (C-2' y C-15'), 31.21 (C-9'), 29.92 (C-5'), 29.85 (C-6'), 29.69 (C-14', C-7', C-4'), 29.50 (C-8' y C-3'), 27.35 (C-10'), 27.06 (C-13'), 22.49 (C-16'), 14.22 (C-17') [6].

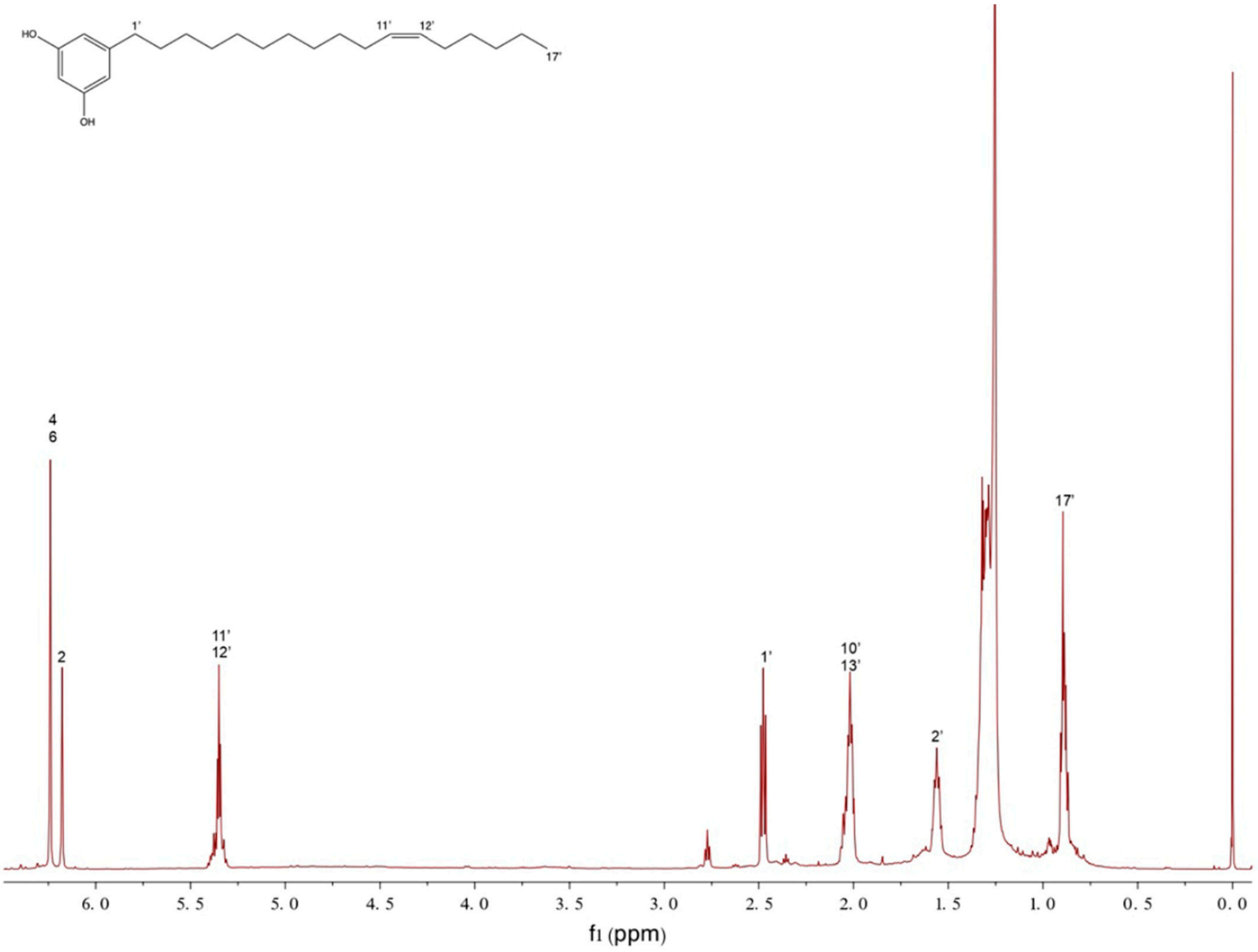

Figure 7. ^{1}H NMR (400 MHz, $CDCl_3$) 5-(11´Z-heptadecenyl)-resorcinol (**7**).

References

1. Shinozaki, J.; Nakane, T.; Onodera, N.; Takano, A.; Masuda, K., Composite constituent: lactucenyl acetate, a novel migrated lupane triterpenoid from Lactuca indica revision of structure of tarolupenyl acetate. *Chem Pharm Bull* **2011,** *59,* 767-769.
2. Viet, T. D.; Xuan, T. D.; Anh, H., α-Amyrin and β-Amyrin Isolated from Celastrus hindsii Leaves and Their Antioxidant, Anti-Xanthine Oxidase, and Anti-Tyrosinase Potentials. *Molecules* **2021,** *26*, 7248
3. Vázquez, L. H.; Palazón, J.; Navarro-Ocaña, A. In 23 The Pentacyclic Triterpenes ,-amyrins : A Review of Sources and Biological Activities, 2018.
4. Maldonado, E.; Diaz-Arumir, H.; Toscano, R. A.; Martinez, M., Lupane Triterpenes with a δ-Lactone at Ring E, from Lippia mexicana. J Nat Prod 2010, 73, 1969-1972.
5. Escobedo-Martinez, C.; Concepcion Lozada, M.; Hernandez-Ortega, S.; Villarreal, M. L.; Gnecco, D.; Enriquez, R. G.; Reynolds, W., 1H and 13C NMR characterization of new cycloartane triterpenes from Mangifera indica. Magn Reson Chem 2012, 50, 52-57.
6. Knoedler, M.; Conrad, J.; Wenzig, E. M.; Bauer, R.; Lacorn, M.; Beifuss, U.; Carle, R.; Schieber, A., Anti-inflammatory 5-(11'Z-heptadecenyl)- and 5-(8'Z,11'Z-heptadecadienyl)-resorcinols from mango (Mangifera indica L.) peels. Phytochem 2008, 69, 988-993.
